# A Nephrologist Perspective on Obesity: From Kidney Injury to Clinical Management

**DOI:** 10.3389/fmed.2021.655871

**Published:** 2021-04-13

**Authors:** Clara García-Carro, Ander Vergara, Sheila Bermejo, María A. Azancot, Joana Sellarés, Maria José Soler

**Affiliations:** ^1^Nephrology Department, San Carlos Clinical University Hospital, Madrid, Spain; ^2^Nephrology Department, Vall d'Hebron University Hospital, Barcelona, Spain; ^3^Nephrology Group, Vall d'Hebron Research Institute, Barcelona, Spain

**Keywords:** obesity, adiposity, chronic kidney disease, insulin resistance, kidney transplantation

## Abstract

Obesity is one of the epidemics of our era. Its prevalence is higher than 30% in the U.S. and it is estimated to increase by 50% in 2030. Obesity is associated with a higher risk of all-cause mortality and it is known to be a cause of chronic kidney disease (CKD). Typically, obesity-related glomerulopathy (ORG) is ascribed to renal hemodynamic changes that lead to hyperfiltration, albuminuria and, finally, impairment in glomerular filtration rate due to glomerulosclerosis. Though not only hemodynamics are responsible for ORG: adipokines could cause local effects on mesangial and tubular cells and podocytes promoting maladaptive responses to hyperfiltration. Furthermore, hypertension and type 2 diabetes mellitus, two conditions generally associated with obesity, are both amplifiers of obesity injury in the renal parenchyma, as well as complications of overweight. As in the native kidney, obesity is also related to worse outcomes in kidney transplantation. Despite its impact in CKD and cardiovascular morbility and mortality, therapeutic strategies to fight against obesity-related CKD were limited for decades to renin-angiotensin blockade and bariatric surgery for patients who accomplished very restrictive criteria. Last years, different drugs have been approved or are under study for the treatment of obesity. Glucagon-like peptide-1 receptor agonists are promising in obesity-related CKD since they have shown benefits in terms of losing weight in obese patients, as well as preventing the onset of macroalbuminuria and slowing the decline of eGFR in type 2 diabetes. These new families of glucose-lowering drugs are a new frontier to be crossed by nephrologists to stop obesity-related CKD progression.

## Introduction

Obesity is a major global public health problem, as the World Health in 1997 (1). Obesity is one of the epidemics of our era. Its prevalence is higher than 30% in the U.S. (WHO 2008) and it is estimated to increase by 50% in 2030. Obesity is defined by excessive body fat accumulation and its incidence is growing, as a result of changes in food habits and physical activity. Recent data indicate there are about 600 million people with obesity worldwide (2) and this number is supposed to increase. Obesity is a chronic and metabolic disease that has an important impact on several specialties such as endocrinology, cardiology and nephrology. As its incidence grows, obesity-related health problems will also be more common in medical specialties.

In Nephrology, obesity-related complications are more than just obese-related glomerulopathy that included glomerulomegaly and focal segmental glomerulosclerosis (FSGS). Several mechanisms have been associated with the obesity-related kidney disease that would mainly be summarized in three groups: the hemodynamic, the adipose tissue-related and the insulin resistance pathways. Increased cardiovascular disease risk and high blood pressure are also health problems associated with obesity that nephrologists have to face. Furthermore, obesity is very common in patients who are in the kidney transplant waiting list, and its presence may be associated with worse allograph transplant function and prognosis. In this article, we will review the main pathophysiological pathways that link obesity and kidney injury, cardiovascular risk and diabetes. In addition, we will also focus on principal strategies in kidney transplantation and pharmaceutical therapies in this population.

## Pathways Involved in Obesity-Related Kidney Disease

Obesity is an independent risk factor for the development of chronic kidney disease (CKD) and it has been related to a decreased glomerular filtration rate (GFR) and albuminuria (3–5). The development of CKD is closely related to other comorbidities that appear in conjunction with weight increase, namely hypertension or insulin-resistance and diabetes. Obese patients with kidney disease show glomerulomegaly and mesangial expansion (6, 7), which are a consequence of the increased blood flow and hyperfiltration that glomeruli suffer. Hyperfiltration in turn produces albuminuria which, together with other damaging mechanisms, leads to a progressive decline in GFR. Therefore, obesity is initially associated with hypertension and hyperfiltration, that produces albuminuria and subsequent loss of GFR.

**Figure 1:**
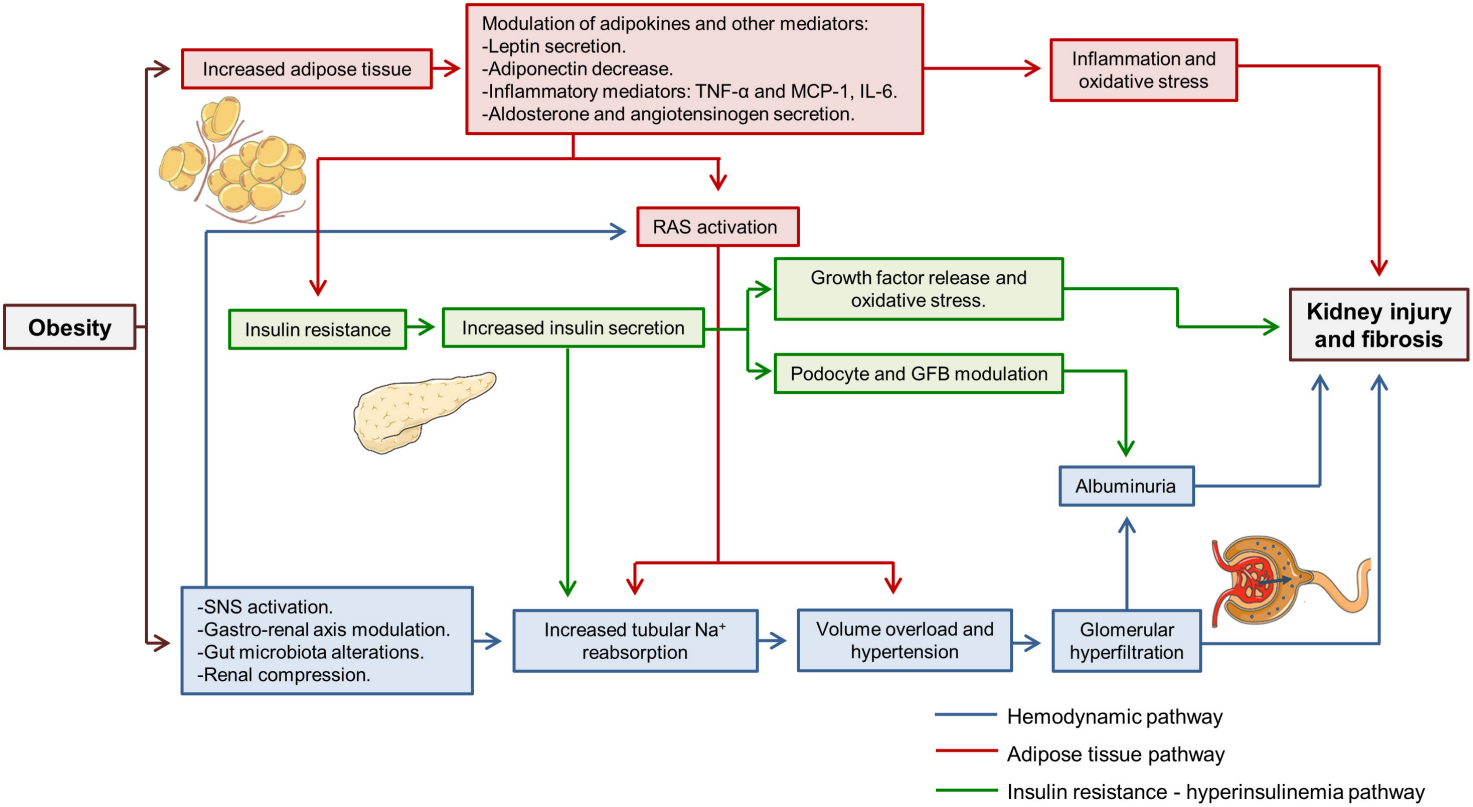
Pathways involved in obesity-related kidney disease. Three main pathways involved (hemodynamic, adipose tissue related and insulin resistance—hyperinsulinemia pathways) have been highlighted in different colors. It can be observed how three pathways interact simultaneously to produce kidney injury. TNF-α, tumor necrosis factor α; MCP-1, monocyte chemoattractant protein 1; IL-6, interleukine 6; RAS, renin-angiotensin system; GFB, glomerular filtration barrier; SNS, sympathetic nervous system; Na^+^, sodium.

Fat mass increase and weight gain activate several harmful pathways that simultaneously damage both the glomeruli and the tubules. Over time, the chronic activation of these pathways leads to progressive kidney injury, the development of albuminuria and CKD (8). Numerous mechanisms have been implied in obesity-related kidney disease, but many of them could be summarized within three main groups: the hemodynamic pathway, the adipose tissue-related pathway and the insulin resistance—hyperinsulinemia pathway (Figure 1). The sole purpose of this last classification is to facilitate the understanding of the mechanisms involved. During the development of kidney injury, the three pathways interact between them and their effect are modulated by other factors associated with obesity and the patient himself, such as diabetes, hypertension, age or sex.

Regarding the hemodynamic pathway, it is proven that obesity increases tubular sodium reabsorption (7, 9). It leads to volume overload and facilitates the development of hypertension, which later contributes to glomerular hyperfiltration and renal microvascular damage. Renin-angiotensin system (RAS) activation is also described in obese patients and animal models (8, 10). RAS activation participates in hypertension development and sodium reabsorption. Moreover, it is a critical step that promotes glomerular hyperfiltration. Some studies postulate that in obesity, sodium reabsorption occurs initially in the first segments of the tubule, namely proximal tubule and the ascending loop of Henle (7, 9). Thus, sodium delivery to distal segments of the tubule is diminished and sensed by macula densa cells, activating tubuloglomerular feedback and inducing renin secretion by juxtaglomerular apparatus (7). This mechanism is similar to that which contributes to hyperfiltration in diabetic kidney disease. Studies performed in 1993 already demonstrated that dogs fed with high-fat diet showed increases renin secretion (9). Different mechanisms have been implied in this initial reabsorption in proximal tubular segments. Obesity induces insulin resistance which produces a compensatory increase in insulin secretion, and the increase in insulin availability has been implicated in increased sodium reabsorption in the proximal tubule and the ascending loop of Henle (9, 11). Recently, the interaction between the gut and the kidney has been described, where secreted gastrin after sodium ingestion acts in the kidney and increases sodium excretion in proximal tubular cells through NHE3 and Na^+^/K^+^-ATPase inhibition (12). In obese patients, an inappropriate diet rich in fats is usually accompanied by increased sodium ingestion, which may interfere with the described gastro-renal axis (8).

The adipose-tissue related pathway is linked to the increase of fat mass that occurs in obese patients. Adipocytes are active cells capable of secreting cytokines named adipokines (13). The increase of adipose tissue modulates adipokine secretion and larger adipocytes are associated with an increased inflammatory adipokine secretion (13). In obesity-related kidney disease, several adipokines have been implied such as leptin, adiponectin, tumor necrosis factor α (TNF-α) or interleukin 6 (IL-6) (7). Leptin secretion by adipose tissue is augmented in obese patients. Leptin has been related to increased activity of the sympathetic nervous system that contributes to hypertension, and leptin infusion in rats raised mean blood pressure (14). In addition, leptin promotes fatty acid oxidation, increasing oxidative stress and the secretion proinflammatory cytokines like monocyte chemoattractant protein 1 (MCP-1) (15). Conversely, adiponectin levels are decreased in obese patients (8). Lower adiponectin levels are associated with insulin resistance and impaired glucose and fatty acids metabolism (13). Besides, adiponectin is involved in glomerular filtration barrier structure regulation. In adiponectin knock-out mice podocyte foot process effacement has been evidenced which leads to an increase in albuminuria, and albuminuria is partially reversed with exogenous adiponectin administration (16). Moreover, in cultured podocytes adiponectin administration reduced permeability to albumin and oxidative stress through AMPK activation (16). Adipose-tissue also regulates RAS. In adipocytes, aldosterone and angiotensinogen secretion have been identified (17). Aldosterone secreted by fat tissue participates in adipocyte differentiation and is implicated in obesity-related hypertension (11, 18).

Insulin resistance is another pathway closely related to obesity-induced kidney disease. Insulin resistance is dependent on the increase in fat mass and the modulation of adipokines that occurs as a consequence of the latter (8). Insulin resistance promotes compensatory insulin secretion and insulin by itself has different effects on the kidney. Insulin acts directly on podocytes through insulin receptors themselves. AKT/mTOR intracellular pathway or glucose transporter 4 (GLUT4) have been related to its signaling (19). Insulin is implied in podocyte function and actin cytoskeleton modulation (20). Thus, augmented insulin secretion affects glomerular filtration barrier selectivity leading to proteinuria. In both rat models and cultured podocytes, insulin administration increases albumin permeability (21). Furthermore, insulin promoted oxidative stress in podocytes through NADPH oxidase activation (21). Insulin also acts on tubules and in cultured renal proximal tubular cells, it promotes TFG-β and collagen IV formation that eventually lead to tubulointerstitial fibrosis (22).

## Obesity and Cardiovascular Disease

Cardiovascular disease (CVD) is an important cause of death and disabilities worldwide. Clinical manifestations of CVD such as acute myocardial infarction, stroke or peripherical vasculopathy are attributed to atherosclerosis. The pathophysiological alterations of atherosclerosis are associated with chronic inflammation of the vessel wall that results from the accumulation of cholesterol-rich atherogenic Apo-B lipoproteins (VLDL, IDL, and LDL) in vascular intima. Lipoprotein accumulation leads to subsequent infiltration of macrophages and T cells of the arterial wall, producing atherosclerotic plaques (23, 24).

Obesity is considered as a major modifiable risk factor for different CVD, even after adjustments for the co-existing risk factors (25). Obesity itself has a deleterious effect on most of the major CVD risk factors: it increases plasma lipid levels, raises blood pressure and impairs glycaemic control in diabetes (26, 27). An increase of body weight, principally abdominal fat accumulation, is associated with a greater atherosclerosis progression. Post-mortem studies have described that both thickness of the adipose panicle and body mass index are associated with more extensive fatty streaks and raised lesions in the right coronary artery (28). In this regard, visceral obesity is also associated with an increased risk for cerebrovascular disease (29). Heart failure (HF) is frequent in obese patients, with approximately 11% of HF in men and 14% in women attributable to obesity (30). It is worth mentioning that an increase of every unit in body mass index (BMI) raises the risk of HF 5% in men and 7% in women (31). Strategies to achieve weight loss in obese patients may be beneficial to reduce the risk of CVD. Among all weight interventions, bariatric surgery has demonstrated beneficial effects on CVD morbidity and mortality (32). Recently, new pharmacologic therapeutics targeting the glucagon-like peptide-1 receptor such as semaglutide are promising for weight loss in adults with obesity or overweight (33).

## Obesity, Inflammation, Lipid Abnormalities, and Hypertension

As mentioned above, one of the mechanisms implied in the increased risk of CVD in obese patients is chronic inflammation driven by the adipose tissue. Adipose tissue is an active endocrine organ capable to synthesize and release into the circulation different types of hormones, peptides, and inflammatory molecules that promote endothelial dysfunction contributing to the development of atherosclerosis (34). Obesity-induced inflammation is marked by an increased infiltration and activation of innate and adaptive immune response. Macrophages are the predominant immune cells infiltrating the adipose tissue in obese individuals. They are polarized into proinflammatory M1 macrophages that secrete proinflammatory cytokines such as TNF-α, interleukins, and C-reactive protein, while simultaneously suppress anti-inflammatory cells and reduce the production of adiponectin, predisposing to increase oxidative stress (35). The inflammatory triggers are still unknown, however, obesity-induced adipose tissue remodeling provides many signals such as adipocyte death, hypoxia and mechanical stress that are capable of initiating an inflammatory response (36).

Lipid abnormalities as elevated triglyceride, VLDL, Apo B, and non-HDL-C levels are typically seen in obesity (37). There is an increase of small dense LDL and it is considered to be more pro-atherogenic than large LDL particles (38). Approximately 60–70% of obese patients are dyslipidemic while 50–60% of patients who are overweight are dyslipidemic (37), increasing risk for cardiovascular disease.

These abnormalities are produced by the greater delivery of free fatty acids to the liver from increased total and visceral adiposity, insulin resistance, and a pro-inflammatory state (37, 39). Adipokines, such as adiponectin and resistin are implicated in lipid metabolism. Levels of adiponectin are decreased in obese patients and are associated with increase in serum triglyceride and decreases in HDL-C levels (40). High levels of resistin have been observed in obesity and it is directly correlated with plasma triglyceride levels (41). Moreover, resistin has been shown to stimulate hepatic VLDL production and secretion due to an increase in the synthesis of Apo B, triglycerides, and cholesterol (41, 42). Finally, resistin is associated with a decrease in HDL-C and Apo A-I levels (42). Additionally, the pro-inflammatory cytokines such as tumoral necrosis factor stimulate lipolysis in adipocytes increasing circulating free fatty acid levels, which increases production of triglyceride by liver and also stimulate *de novo* production of VLDL and triglycerides (43). At higher levels the pro-inflammatory cytokines decrease the expression of lipoprotein lipase and increase the expression of angiopoietin like protein 4, an inhibitor of lipoprotein lipase (43, 44), delaying the clearance of triglyceride rich lipoproteins. Pro-inflammatory cytokines also affect HDL metabolism, decreasing the production of Apo A-I, the main protein constituent of HDL. These changes induced by pro-inflammatory cytokines result in a decrease in reverse cholesterol transport that plays a key role in preventing cholesterol accumulation in macrophages thereby reducing atherosclerosis (45).

Hypertension is frequent in obesity (46), and the mechanisms implicated are under investigation. Endothelial disfunction constitutes the initial alteration that initiates atherosclerotic disease. In this regard, it has been previously described in obese children and adolescents, that the number of circulating endothelial cells, a type of cells that reflect endothelial damage, are related with greater body fat percentage and hypertension. This suggests an association between body fat, endothelial dysfunction and hypertension (47). Obesity-induced inflammation, insulin resistance and high plasma leptin promote an impairment of synthesis of nitric oxide (NO) producing an imbalance between vasodilatation and vasoconstriction (48), favoring arterial stiffness and vascular remodeling what promotes arterial hypertension. It has also been observed that sympathetic activation plays an important role in obese-related hypertension. High intake of fat stimulates peripheral α1 and β-adrenergic receptors, increasing sympathetic activity and hypertension (49, 50). Moreover, parasympathetic activity is also altered, probably related to the presence of atherosclerotic plaque in large arteries and an increase of central stiffness impairing the baroreflex sensitivity (46). High levels of plasma renin activity, plasma angiotensin, angiotensin II and aldosterone are also associated with obesity (51–53). Renin, angiotensin II, angiotensinogen and angiotensin II receptors are found in AT suggesting that RAS is settled at adipocyte cells (54). RAS activation leads in turn to a salt-sensitive hypertension and vasoconstriction.

In this context, obesity-related hypertension treatment should be multidisciplinary. The treatment program should include an important lifestyle intervention that includes a low caloric diet with low intake of fat, increase of physical activity, reduced salt intake and pharmacological treatment (55). The first option of antihypertensive drugs in obese patients are angiotensin receptors blockers (ARB) and angiotensin converting enzyme inhibitors (ACEi) because both are associated with a lower incidence of diabetes (56), favorable effects on left ventricular hypertrophy and reduce risk of obese-related glomerulopathy.

## Intersection Between Diabetes and Obesity: Diabesity

The term “diabesity” was first described in the 1970s to name the strong relationship that exists between these two entities, diabetes and obesity. Both of them, together with high blood pressure and dyslipidemia, constitute the cluster of conditions known as “metabolic syndrome,” which is associated with a higher CVD risk (57). In addition, both obesity and diabetes are increasing their prevalence worldwide (58), mainly explained by changes in lifestyle and by genetic susceptibility (2).

**Figure 2:**
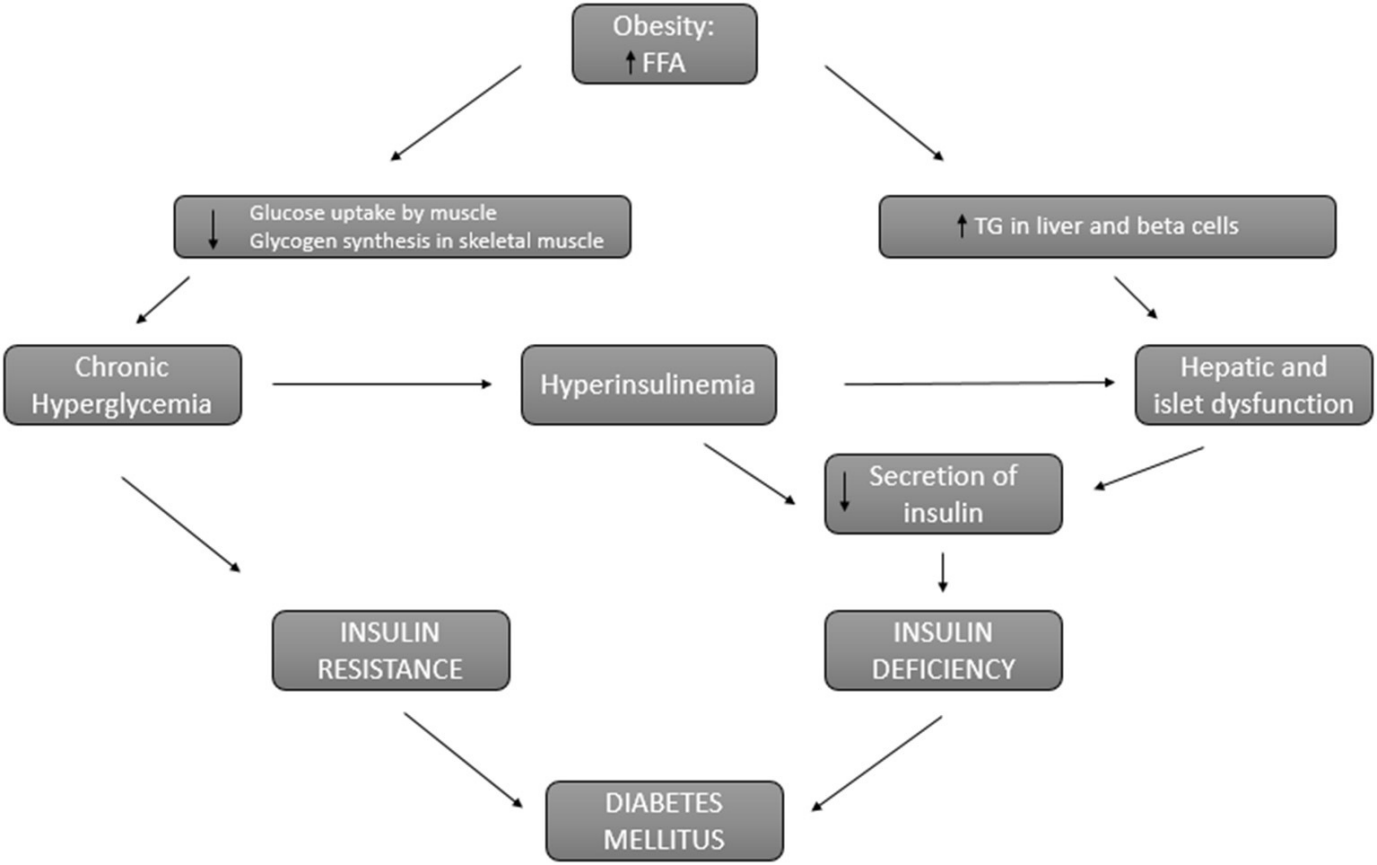
Connection between obesity and diabetes. Pathways involved in insulin resistance and insulin deficiency that lead to diabetes in obese patients. FFA, free fatty acids; TG, triglycerides.

It is known that overweight and obesity increase the risk of type 2 diabetes (59, 60). This risk is proportional to body mass index increase and it is higher in the presence of abdominal fat. This abdominal fat is related to insulin resistance and has been identified as an independent risk factor for the development of type 2 diabetes in obese patients (61). In fact, the pathophysiology that connects obesity and diabetes is based on two main factors: insulin resistance and insulin deficiency (62) (Figure 2). In obese patients, circulatory levels of free fatty acids are increased, inhibiting glucose transport and promoting the use of lipids by the muscle, which causes insulin resistance. On the other hand, the decrease in glucose use by the muscle develops chronic hyperglycemia that perpetuates insulin resistance and in turn promotes increased insulin secretion. This hyperinsulinemia is also perpetuated due to the lipotoxicity of the liver cells derived from the accumulation of lipids in the liver and pancreatic β-cells rather than obesity itself (63). Thus, hyperinsulinemia and liver and pancreatic lipotoxicity promote an insulin deficit. Furthermore, obese patients present a systemic pro-inflammatory state that promotes insulin resistance leading to established diabetes along with insulin deficiency (64).

Interestingly, there are certain abnormalities in metabolism that play a role in the development of diabetes and obesity. It has been shown that oxidative stress, inflammation and advanced glycation end-products can induce microvascular damage and a deficit in tissue perfusion (65). Epidemiological studies have found an association between short periods of sleep and increased diabetes or obesity. This lack of sleep has been linked to a decrease in leptin and an increase of hunger and intake of carbohydrates and food with high quantity of calories (66). It has been also demonstrated that hormones, namely testosterone, also play a role in diabesity. In men, an inverse relationship has been evidenced between testosterone concentration and visceral fat accumulation. In women, an increase in testosterone has been associated with glucose intolerance and insulin resistance (67). Vitamin D has been reported to inhibit fat accumulation, increase insulin secretion, preserve pancreatic β-cells, decrease insulin resistance and reduce appetite (68). Finally, the gut microbiota also contributes to the development of diabetes and obesity: a reduction in Bacteroidetes and an increase in Firmicutes has been associated with obesity (69).

In relation to DM, obesity and their common pathways it is important to mention sodium-glucose cotransporters (SGLT), that are widely distributed in the body, especially in the renal tubules, where glucose is reabsorbed. There are two subtypes of these cotransporters: SGLT-1 and SGLT-2. Most of the glucose is reabsorbed by the SGLT-2. In patients with DM there is an over expression of SGLT-2 in the hyperfiltration phase (70). Nowadays, exists a newly line of drugs to inhibit these SGLT2, increasing the excretion of glucose through the urine in these patients. Thus, the glycemic control improves with a reduction in glycosylated hemoglobin of 0.66%, weight loss of 1.8 kg and a decrease in systolic blood pressure of 4.45 mmHg compared to placebo (71). These benefits are promising in diabesity patients. In addition, beneficial results of said treatment have been shown in terms of cardiovascular mortality and morbidity and improvement in renal prognosis. One of the most frequently observed adverse effects is an increase in urinary tract infections and fungal genital infections derived from increased glycosuria (72).

## Impact of Obesity Before and After Kidney Transplantation

Obesity is not considered to be a contraindication for kidney transplantation according to most clinical practice guidelines (73, 74). However, the reality is that many centers avoid transplanting obese patients (75), considering a BMI>35 Kg/m^2^ a relative contraindication to kidney transplantation (76). Once on the waiting list, obese patients experience longer waiting times and fewer chances to access to a transplant compared to non-obese patients (77, 78). In a large population-based study using the United Network for Organ Sharing (UNOS) data on 132,353 patients on the waiting list, revealed that the probability of receiving kidney transplantation decreased with the increase of BMI. Furthermore, obese patients were more likely excluded when an allograft was available as the BMI increased (79). Importantly, evidence shows that kidney transplantation in obese patients offers better survival than remaining on dialysis. In a registry study using the United States Renal Data System (USRDS) data in which 7,443 obese patients were included, the mortality rate for those who received a kidney transplant was half of those who stayed on the waiting list (3.3 deaths vs. 6.6 deaths per 100 patients-year), and the mortality rate was even lower in the subgroup of patients who received a living donor transplant. However, this survival advantage was not achieved on patients with BMI ≥40 Kg/m^2^(80). In a more recent study using also the USRDS data that included 208.986 patients, a survival benefit was reported in all grades of obesity for both living and deceased donor kidney transplant recipients, although the benefit observed was lower in patients with BMI ≥ 40 Kg/m^2^ (78). In this study, better patient survival was also demonstrated in the subgroup of patients who received a kidney from an expanded criteria donor, and the only subgroup in which a survival benefit could not be demonstrated were Black patients with BMI ≥ 40 Kg/m^2^.

There are conflicting results regarding the association between patient and graft survival with obesity. While there are studies that have shown a negative impact of obesity on both patient and graft survival (81–85), others have found no association (86–89). In a systematic review and meta-analyses that included 138,081 kidney transplant recipients, obesity was associated with increased graft failure but no differences were found between obese and non-obese patients in terms of survival (90). A large registry study that included more than 50,000 patients, reported worse graft and patient outcomes in patients with very low and very high BMI (82). A recent systematic review that included data from more than 200,000 kidney transplant recipients found worse patient and graft survival at one, two and three years post-transplant in obese compared to non-obese patients (91). In contrast, the ANZDATA registry-based study (*n* = 5,864) did not show an independent association between obesity and patient or graft survival, although obese patients were more likely to experience delayed graft function (DGF) and acute rejection (AR) (92). Other single-center retrospective studies have not shown any impact on patient survival or graft loss when comparing outcomes between obese and non-obese patients (88, 93, 94). Interestingly, in a single-center retrospective study, patient and graft survival in obese patients was comparable to non-obese patients when those suffering from surgical wound complications were excluded from the analysis (95).

Delayed graft function is associated with recipient BMI in many retrospective studies (89, 91, 92, 96), and a UNOS registry-based study showed a significant gradual increase in the risk of DGF according to the obesity grade (83). The association between obesity and rejection is controversial. Some studies have shown an increased risk of rejection in this population (85, 87, 92, 94, 97), while others have not (86, 98–101). There is an intrinsic problem in obese patients regarding the difficulty on achieving and adequate exposure to immunosuppression, with frequent overexposure resulting into calcineurin-inhibitor toxicity, or otherwise, underexposure, placing these patients at a higher risk of rejection (99). Surgical interventions in obese patients are complex, technically more difficult and result in an increased risk of surgical complications (91, 95, 100, 102). Obese patients are more likely to have an increased risk for surgical wound infections, which have been reported to range from 20–30% in patients with BMI 30–40Kg/m^2^, and up to 40% in patients with BMI ≥ 40 Kg/m^2^ (95, 103). Reducing surgical complications becomes important if we consider that there is evidence showing that outcomes are similar between obese and non-obese patients in the absence of such complications (95).

Weight gain after transplantation is very common (81, 104), and a significant increase in weight after transplantation has been associated with decreased patient survival (81). Obesity has also been associated with an increased risk of cardiac events and congestive heart failure after transplantation (105), as well as with the development of diabetes mellitus (106, 107). Although there is conflicting data regarding the impact of obesity on kidney transplantation, it undoubtedly represents a risk factor for surgical complications, and it probably has a real impact on outcomes. Obese patients cannot be excluded from transplantation because of their BMI, since survival after transplantation is better than remaining on dialysis. Thus, management of these patients must include multidisciplinary strategies to lose weight, including the possibility of bariatric surgery in selected candidates as well as pharmacological treatments (85, 108).

## Pharmacological Management of Obesity

The management of a patient with obesity must be holistic. Medical treatments are only a piece in a puzzle that includes physical activity, nutrition, behavioral therapy, pharmacological treatment, and, sometimes, bariatric surgery (109). Thus, anti-obesity pharmacological therapies should always be established together with nutritional and behavioral assessment and physical activity advice. Generally, anti-obesity drugs are prescribed by qualified clinicians in obesity units. However, some of them are commonly used in nephrological patients, since they are not obesity-exclusive treatments. Pharmacological management should be quickly considered in patients who do not lose weight with lifestyle modifications or are not able to maintain weight reductions.

At the time this article is written, four drugs have been approved for long-term (>12 weeks) obesity treatment by the U.S. Food and Drug Administration (FDA): orlistat, naltrexone extended-release (ER)/bupropion ER, phentermine/topiramate controlled-release (CR), and liraglutide (110). Phentermine/topiramate has not been approved for long-term use by the European Medicines Agency (110). Recommendations for starting anti-obesity drugs differ from U.S. and Asian guidelines: in the U.S. are recommended for patients with BMI ≥30 or ≥27 kg/m^2^ with comorbidities (diabetes, hypertension, dyslipidemia, sleep apnea) (111) while in Asia are recommended with lower BMI (≥25 or ≥23 kg/m^2^) to patients who present at least one weight-related comorbidity (1).

Orlistat works as a gastrointestinal lipase inhibitor, avoiding fatty acids intestinal absorption and weight loss is about 5% (112, 113). Due to its mechanism of action, flatus is an important side effect, but orlistat has been also related to malabsorption, especially of fat-soluble vitamins. It has been associated with kidney stones and contraindications include malabsorption syndrome and cholestasis. Pancreatitis and liver disease are very rare. Data on patients with CKD are scarce. A study including 32 patients with CKD stages 3–5 showed significant weight loss and no serious adverse events in this population (75). Furthermore, Son JW et al. recommended that both orlistat and liraglutide can be carefully used in CKD patients with obesity (114).

Liraglutide is a glucagon-like peptide-1 (GLP-1) receptor agonist that was firstly approved for the treatment of type 2 diabetes. It is 97% similar to human GLP-1 but it has a longer action time (115). It works by reducing appetite, delaying gastric emptying and balancing insulin and glucagon secretion time (115). At the highest dose of 3 mg per day, liraglutide has shown benefits in obese patients, with 5–10% body weight loss and an increased time of onset of diabetes in prediabetic obese patients (116). Liraglutide has also shown benefits in terms of glycemic control, blood pressure, lipid levels (116). The dose of 1.8 mg has been related to a decreased risk of death from cardiovascular disease, non-fatal myocardial infarction, and non-fatal cerebral infarction in diabetic patients (117). Main side effects are gastrointestinal: nausea and vomiting, constipation and diarrhea. Slowly increasing of dose could help to diminish them (117). Liraglutide has also been associated with pancreatitis and should not be used in patients with a previous history of pancreatitis (118). It also should not be used in patients with a personal or family history of medullar thyroid cancer or multiple endocrine neoplasia (119).

Bupropion is used for smoking cessation and depression. It inhibits appetite *via* the reward system and increases energy consumption. Naltrexone is an opioid antagonist that also inhibits food intake. Their combination has a synergistic effect (120). Weight reduction seems to depend on the dose: 6.1% for naltrexone/bupropion 32/360 mg and 5% for the 16/360 mg group according to the Contrave Obesity Research (COR)-I trial. The combination of both drugs has shown improvement in glycemic control, insulin resistance, and lipid profiles (121). The main side effect is nausea, which makes that some patients discontinue the treatment. Emotional or psychological disorders could appear in patients without psychiatric disease and the risk of suicidal ideation in young patients taking bupropion has been reported by FDA. Combination of naltrexone/bupropion could increase the risk of seizures, especially in patients with a previous history or with excessive alcohol intake or cocaine, as FDA notes. This drug should not be used in patients with end-stage CKD, since there are no studies in this population.

Combination of phentermine (sympathomimetic amine, anti-obesity drug) and topiramate (used to treat seizures and migraine headaches) drives weight reduction through increased satiety and energy waste, reducing caloric intake and taste abnormalities (122). Weight loss is about 5–10%. As well as other anti-obesity drugs, it has shown improvements in cardiovascular risk factors (123). A metanalysis recently published showed that phentermine/topiramate has the greatest weight loss effect among the currently available anti-obesity drugs (124). A pregnancy test must be performed before starting phentermine/topiramate since topiramate can cause birth defects. It also could cause depression, anxiety, sleep disorder, suicidal ideation, and difficulties in concentration. Topiramate has been related to inhibition of carbonic anhydrase activity and subsequently, metabolic acidosis, hypokalemia, renal stones, angle-closure glaucoma could be side effects. In patients with CKD this treatment should be avoided. Of note that this drug has not been approved for obesity treatment in Europe.

As Son JW et al. mentioned, the weight loss effect of these anti-obesity drugs can be ranked as follows: phentermine/topiramate CR > liraglutide 3.0 mg > naltrexone ER/bupropion ER > orlistat. New clinical trials are ongoing to study new anti-obesity drugs, such as sodium-glucose cotransporter two inhibitors, other GLP-1 receptor agonists, dopamine reuptake inhibitors or mineralocorticoid receptor agonists (114). Obesity therapies goal is to treat adipose tissue dysfunction, excessive body fat and diseases that are secondary to increased body fat and its adverse consequences, as renal impairment. It is worth mentioning that some drugs that are frequently used by nephrologists are also associated to body weight gain, such as insulin (125), glucocorticoids (126) and some β-blockers (atenolol, metoprolol) (127).

## Conclusions

We reviewed the obesity-related kidney disease regarding the involved pathophysiologic mechanisms that include the hemodynamic, the adipose tissue-related and the insulin resistance—hyperinsulinemia pathways. Advances on the knowledge of the link between obesity, cardiovascular risk and diabetes in CKD patients including the crucial importance of obesity pre/post kidney transplantation are of great interest for the management of renal patients with obesity. The discussed studies provide insights on new frontiers regarding the fact that new pharmacological strategies are promising in a future for treating and management of obesity.

## Author Contributions

CG-C, AV, SB, MA, JS, and MS have conceptualized and written the manuscript. All authors contributed to the article and approved the submitted version.

## Conflict of Interest

AV reports non-financial support from Mundipharma, outside the submitted work. MS reports personal fees from NovoNordisk, Janssen, AstraZeneca, Fresenius, Mundipharma, Pfizer, Bayer and Vifor, grants and non-financial support from Boehringer, and non-financial support from Eli Lilly and Esteve outside of the submitted work. CG-C has received travel and congress fees support from Astra-Zeneca, Esteve, NovoNordisk, Boehringer-Ingelheim Lilly, Astellas, Otsuka, Novartis and Baxter. CG-C has given scientific lectures and participated in advisory boards organized by Astra-Zeneca, Boehringer-Ingelheim Lilly, Mundipharma and NovoNordisk. The remaining authors declare that the research was conducted in the absence of any commercial or financial relationships that could be construed as a potential conflict of interest.
